# Decoupling speed and accuracy in an urgent decision-making task reveals multiple contributions to their trade-off

**DOI:** 10.3389/fnins.2014.00085

**Published:** 2014-04-23

**Authors:** Emilio Salinas, Veronica E. Scerra, Christopher K. Hauser, M. Gabriela Costello, Terrence R. Stanford

**Affiliations:** Department of Neurobiology and Anatomy, Wake Forest School of MedicineWinston-Salem, NC, USA

**Keywords:** choice, computational model, decision making, discrimination, mental chronometry, race to threshold, saccade, subtraction method

## Abstract

A key goal in the study of decision making is determining how neural networks involved in perception and motor planning interact to generate a given choice, but this is complicated due to the internal trade-off between speed and accuracy, which confounds their individual contributions. Urgent decisions, however, are special: they may range between random and fully informed, depending on the amount of processing time (or stimulus viewing time) available in each trial, but regardless, movement preparation always starts early on. As a consequence, under time pressure it is possible to produce a psychophysical curve that characterizes perceptual performance independently of reaction time, and this, in turn, makes it possible to pinpoint how perceptual information (which requires sensory input) modulates motor planning (which does not) to guide a choice. Here we review experiments in which, on the basis of this approach, the origin of the speed-accuracy trade-off becomes particularly transparent. Psychophysical, neurophysiological, and modeling results in the “compelled-saccade” task indicate that, during urgent decision making, perceptual information—if and whenever it becomes available—accelerates or decelerates competing motor plans that are already ongoing. This interaction affects both the reaction time and the probability of success in any given trial. In two experiments with reward asymmetries, we find that speed and accuracy can be traded in different amounts and for different reasons, depending on how the particular task contingencies affect specific neural mechanisms related to perception and motor planning. Therefore, from the vantage point of urgent decisions, the speed-accuracy trade-off is not a unique phenomenon tied to a single underlying mechanism, but rather a typical outcome of many possible combinations of internal adjustments within sensory-motor neural circuits.

## 1. The problem of parsing the reaction time

In daily life, some decisions are rather abstract (should I trust the financial adviser?) whereas others require a specific action (should I press the brake or the accelerator?). Within the latter category, speed and accuracy are inversely related in virtually every task (Woodworth, 1899; Hick, 1952; Wickelgren, 1977; Chittka et al., 2009); the faster the decision, the less accurate the outcome. This means that the traditional, key quantities that are used to measure cognitive performance, the reaction time (**RT**) and the percentage of correct responses, are fundamentally intertwined. What is the underlying cause of this interdependence? How does it emerge from the structure and dynamics of neural circuits? We consider these questions in the context of decisions that are coupled to immediate actions.

Intuitive models of the speed-accuracy trade-off have been formulated (Reddi and Carpenter, 2000; Bogacz et al., 2010; Standage et al., 2013), but the empirical investigation of these questions reveals further complexity (Cook and Maunsell, 2002; DiCarlo and Maunsell, 2005; Battaglia and Schrater, 2007; Cohen et al., 2009; Heitz and Schall, 2012). Part of the problem is that the RT reflects the total amount of time consumed by all the subsystems that contribute to a choice or decision process. Thus, when a subject executes an action in response to a sensory scene, the RT must comprise, at the very least, the time necessary for analyzing the sensory information plus the amount of time required to plan the motor action that is congruent with that information. Discerning just these two components has been challenging because the underlying neural networks are themselves strongly interrelated: neurons that encode a subject's perceptual decision, that participate in motor planning, or that do both, are typically found within the same, local microcircuits (Horwitz and Newsome, 1999; Shadlen and Newsome, 2001; Hernández et al., 2010; Costello et al., 2013; Mante et al., 2013). Furthermore, other distinct cognitive processes may contribute to the RT too; for instance, deploying visuospatial attention or accessing information stored in memory could represent separate processing steps requiring a certain amount of time to unfold independently of the perceptual and motor-planning stages (Sternberg, 1966; Monsell, 2003; Horowitz et al., 2004; Busse et al., 2008). As such, the RT must reflect a total sum over the times consumed by multiple covert processes (Sternberg, 1969), each of which could conceivably constrain or be traded against the others.

Broadly speaking, three techniques have been used to distinguish the two major components of the RT during relatively fast perceptual decision-making tasks, i.e., the contributions of perceptual and motor-planning processes. (1) A common approach is to introduce a delay between the perceptual evaluation and the motor report required in each trial. This strategy is widely used to characterize neuronal activity as sensory-, memory-, or movement-related neurons (Shadlen and Newsome, 2001; Sommer and Wurtz, 2001; Lemus et al., 2007). (2) Another possibility is to limit the amount of cue viewing time (Bergen and Julesz, 1983; Ratcliff and Rouder, 2000; Bodelón et al., 2007; Kiani et al., 2008). The idea is that neurons, or any processing component in general, whose responses vary systematically as functions of cue viewing time may be strongly involved in the analysis of perceptual information. This manipulation is not as straightforward as it may seem, though, because controlling very short stimulus durations is difficult and typically requires additional masking stimuli to prevent stimulus persistence, and such masking introduces other potential problems (Breitmeyer and Ogmen, 2000, 2006). (3) An alternative that is not quite as intuitive, is to do the reverse of 1: inform choices on the basis of urgent perceptual decisions. That is, start preparing a motor response first, before the relevant cue information becomes available (Ghez et al., 1989; Chapman et al., 2010). That way, the initial motor planning stage stays relatively constant.

That is the approach we have taken (Stanford et al., 2010; Shankar et al., 2011; Costello et al., 2013). It provides a simple and highly effective way to dissociate motor and perceptual performance, and thus a different set of tools with which to characterize and quantify perceptual decision-making mechanisms. Here we review previously published results of experiments in which urgent decisions inform rapid choices (Stanford et al., 2010; Shankar et al., 2011; Costello et al., 2013), but focus specifically on their implications for understanding the origin of the speed-accuracy trade-off. As discussed below, under this light it is possible to see not only how perceptual capacity and motor execution interact to determine the response speed and success rate of a subject, but also how additional factors such as motivation or internal preference may alter that interaction. In this way, it becomes quite clear that the speed-accuracy trade-off is not a unitary phenomenon derived from a unique, underlying mechanism, but is instead the result of multiple, semi-independent moving parts that interact with each other within sensory-motor neural circuits.

## 2. Perceptual decisions under time pressure

**Figure 1:**
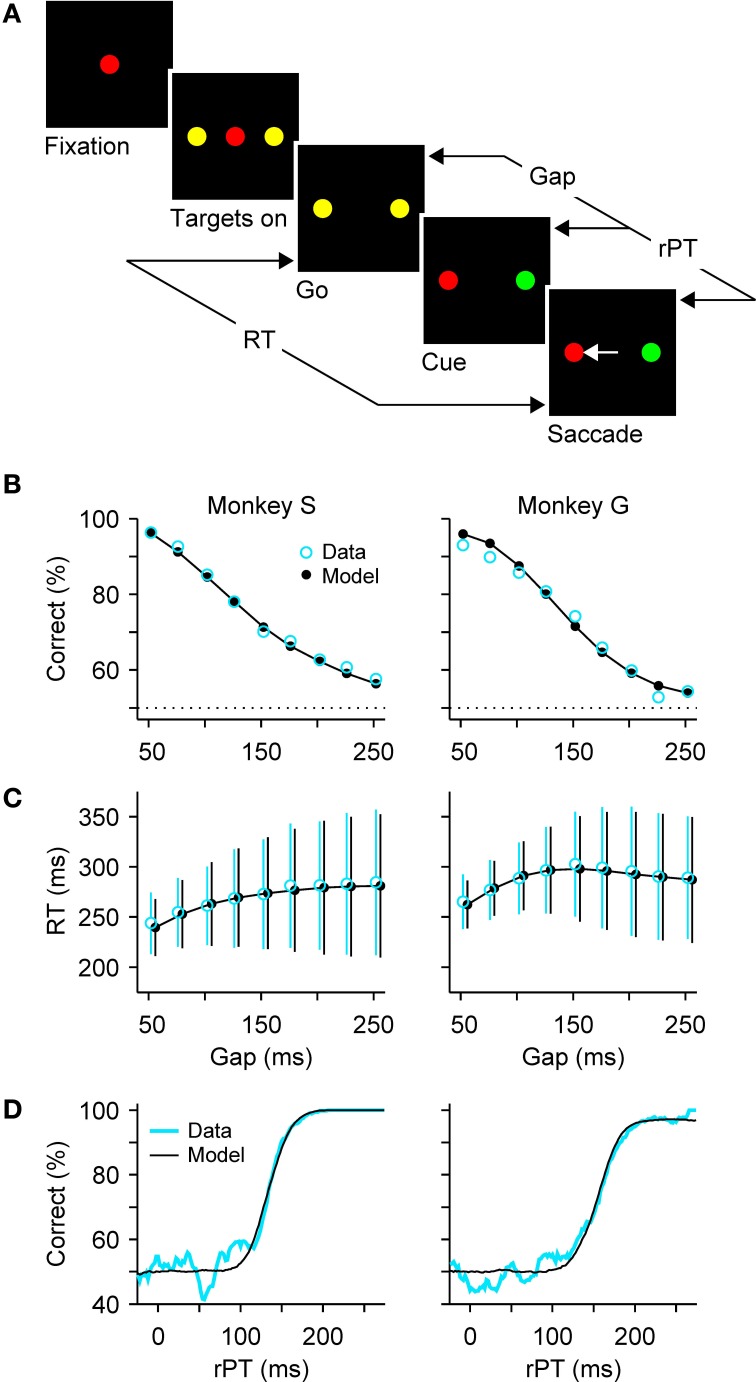
**Dissociating perceptual and motor performance in the compelled-saccade task**. **(A)** Sequence of events in the task. The subject is required to make a saccade when the fixation point disappears (go). If the chosen target matches the color of the fixation point (red, in this example), the choice is correct and a reward is obtained. The go instruction is given first, before the relevant sensory information is revealed (cue). The gap (50—250 ms) is the time interval between the go and the cue. The rPT is the amount of time during which the color information can potentially inform the saccadic choice. **(B)** Percentage of correct responses as a function of gap, or psychometric curve. **(C)** Mean RT (±1 SD) as a function of gap, or chronometric curve. Both correct and incorrect trials are included. (**D**) Percentage of correct responses as a function of rPT (equal to RT − gap), or tachometric curve. In **(B–D)**, blue and black lines/symbols correspond to psychophysical and simulation results, respectively. See Shankar et al. (2011) for details about the experimental data and modeling methods.

As a means to disambiguate perceptual and motor processes, we designed a compelled-response task wherein participants are given the instruction to respond *before* the relevant perceptual information appears (Stanford et al., 2010). In the oculomotor version, the compelled-saccade task (Figure 1A), the response is an eye movement. First, the observer fixates on a central spot, the color of which indicates the color of the eventual target. Then two yellow (neutral) dots appear in the periphery; these are simply placeholders indicating the possible response locations. Next, the central fixation point disappears, and this is the “go” signal that tells the observer “respond now!” Note that, when the go is given, the identities of the target and distracter are still unknown, but the observer must begin planning a movement to one of the two potential targets nonetheless. Then, after a variable time gap (from 50 to 250 ms) the peripheral dots change color, revealing one to be the target and the other the distracter. The onset of the subject's response occurs when the eyes just start moving, and marks the end of the RT period that started at the go signal (Figure 1A).

The logic behind this design is that, by telling the subject when to respond, the motor choice process is initiated early, and so perceptual information, once presented, influences a motor plan that is already developing. By unpredictably varying the time delay between the go signal and the appearance of the color cue (i.e., the gap), the subject generates responses that range between fully informed choices (for gaps that are much shorter than the typical saccadic RT) and fully uninformed choices, or guesses (for gaps that are comparable to the typical saccadic RT), all with the same underlying distribution of motor plans. So, it becomes possible to dissociate the effect of motor preparation from the perceptual decision-making process in an otherwise standard saccadic choice task.

The crucial event in the task is the go instruction, which compels the subject to respond before the target and distracter are revealed. But, why is it that subjects do not simply wait for the color cue to appear before making a choice? In essence, there are three reasons. First, because responding is natural; with the fixation point gone and two salient objects present, it takes effort not to look at one of them. Second, because throughout training, the subjects learn two separate rules, (1) that the offset of the fixation point means “respond now!” and (2) that the correct choice is the one matching the color of the fixation point. Rule 1 is learned first, and if necessary, which is not always the case, it is practiced independently of rule 2. And third, during the compelled-saccade task subjects have a limited time window for making a valid response, so a trial is scored as incorrect—and no reward is given—if the RT is too long, regardless of the choice. It should be noted, however, that consistent with the first two points, such trials in which the RT limit is exceeded are extremely rare (<2%). For a detailed analysis of possible waiting strategies see Salinas et al. (2010).

Performance in the task is expected to decline toward chance as a function of the gap, and indeed this is what happens, as illustrated with representative data from two monkeys (Figure 1B). In contrast, RTs are expected to remain approximately—but not exactly—constant, and this is also the case: mean RTs change by less than 30 ms, or approximately 10%, while performance varies between chance and near 100% correct (Figure 1C). In other perceptual decision-making tasks, RTs often show comparable variations of a few tens of milliseconds, although the difference over the full performance range sometimes reaches hundreds of milliseconds, or several fold (Wolfe, 1998; Ratcliff and Smith, 2004; Palmer et al., 2005; Reinagel, 2013a,b). The variation of ~30 ms in mean RT seen in the compelled-saccade task is modest, but more importantly, it and the systematic increase in the spread of the RTs with gap (error bars in Figure 1C) can be fully accounted for by a simple model; the key notion is that even though the initial motor planning process is statistically the same for all the gaps, the motor conflict is resolved sooner or later depending on when the perceptual information arrives (more on this below).

### 2.1. The tachometric curve

Although the gap is the main control parameter in the task, the variable that fundamentally determines the probability of success in each trial is the raw processing time, or **rPT** (Figure 1A), which is the amount of time before the onset of the motor response during which the color information is available to inform the choice. It is important to stress that this theoretical limit to the maximum amount of cue viewing time is a trial-specific quantity, which can be easily computed via

(1) rPT=RT−gap

based on the gap and the RT recorded in each trial. Using this equation we can determine how long the perceptual information was available for guiding each saccadic choice. Furthermore, by plotting the percentage of correct responses versus rPT we obtain a “tachometric curve,” a curve that characterizes the perceptual performance of a subject (Figure 1D).

This curve has a sigmoidal shape with parameters that are readily interpretable in terms of psychophysical capacity. For the data in Figure 1D, the saturation values are very near 100% correct, so the color information is fully exploited by the subjects when they have enough time to view the cue. The center point of the curve is the rPT at which the percent correct is halfway between chance and the saturation value. It is an indication of how much viewing time is necessary for perceptual information to have a significant impact on performance. For the curves in Figure 1D, the center points are 134 ± 2 ms for monkey S and 157 ± 2 ms for monkey G. Trials to the left of the center point are mostly near chance performance and correspond predominantly to uninformed choices, or guesses, whereas trials to the right of the center point correspond mostly to fully informed choices. Finally, the steepness of the curve near the center point provides a measure of the speed with which perceptual information influences the choice once this information has begun having an impact. For monkeys S and G, half of the performance range (from 62.5% to 87.5% correct) is covered within 24 ± 2 ms and 40 ± 2 ms, respectively, so the color discrimination unfolds extremely rapidly once it gets going.

### 2.2. Multiple mechanisms for generating trade-offs

In principle, simultaneous variations in RT and percent correct may result from changes in motor planning alone, in perception alone, or in both, and to distinguish these options it is essential to have independent, quantifiable measures of their impact on choice behavior. That is the key advantage of the compelled-response approach, it provides independent assessments of perceptual and motor performance in the tachometric and chronometric (RT versus gap) curves, respectively. This is illustrated in detail further below with data from two experiments, but before discussing those, it is useful, first, to consider some simplified examples, and second, to gain some mechanistic intuition, via a heuristic model, about the ways in which perceptual and motor-planning processes interact when decisions are made under time pressure. The three scenarios that follow are meant simply to illustrate, based on the model, how variations in the three psychophysical curves obtained in the compelled-saccade task (Figures 1B–D) may relate to each other.

**Figure 2:**
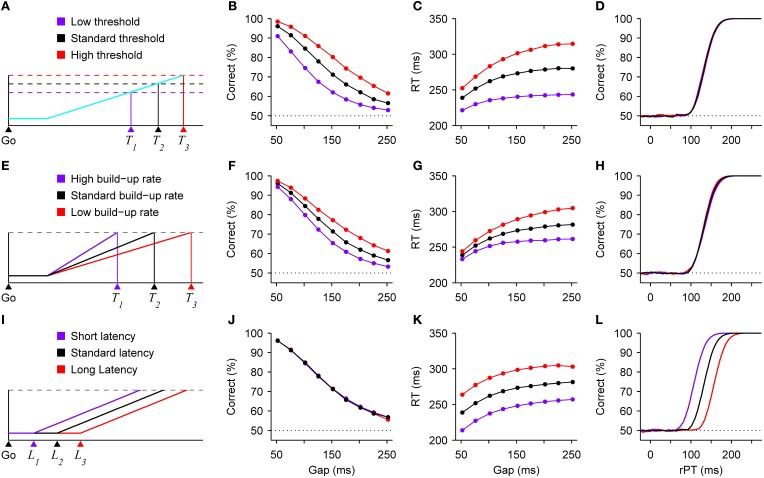
**Changes in speed and accuracy generated by three distinct mechanisms**. All results are expectations based on model simulations of the compelled-saccade task. **(A)** Schematic of a developing oculomotor plan. A saccade is triggered shortly after motor-related activity (blue trace) reaches a threshold (dotted lines). The threshold-crossing time, and thus the RT, varies with the threshold value. **(B–D)** Model results. Variations in threshold produce a standard speed-accuracy trade-off. As the threshold increases, both performance **(B)** and mean RT **(C)** increase, but the tachometric curve **(D)** does not change. **(E)** Schematic illustrating how threshold-crossing time varies with the build-up rate of the motor plan. **(F–H)** Model results. Variations in mean build-up rate also produce a trade-off. As the mean build-up rate decreases, both performance **(F)** and mean RT **(G)** increase, whereas the tachometric curve **(H)** changes minimally. **(I)** Schematic illustrating how threshold-crossing time varies with the latency of the go signal. **(J–L)** Model results. Variations in visual latency alone do not produce a trade-off. When the latency of the visual stimuli (go signal and color cue) decreases, performance does not change **(E)**, but mean RT **(F)** decreases and the tachometric curve **(G)** shifts to the left, indicating that perception informs the subject's choices systematically sooner. Results are from model simulations either with identical parameters as in Figure 1 (for monkey S), or with a 25% increase or a 25% decrease in the value of one parameter, either the threshold **(B–D)**, the mean build-up rate of the motor plans **(F–H)**, or the latency of the visual information **(J–L)**.

The speed-accuracy trade-off is often explained in terms of a change in threshold (Reddi and Carpenter, 2000; Bogacz et al., 2010; Hanks et al., 2011). That is, a motor response is triggered after a “decision variable” reaches a particular value (Figure 2A), and increasing that value produces both higher RTs and a higher proportion of correct responses. This is because it takes longer for the variable to go from baseline to threshold in each trial, and a longer RT means more time during which the perceptual information can advance the decision variable in the correct direction. Although our theoretical framework is somewhat different (and considers the threshold to be fixed; see below), in the compelled-saccade task a change in threshold would have precisely the expected effects associated with a standard trade-off (Figures 2B,C), but notably, it would have absolutely no impact on the tachometric curve (Figure 2D). This is because, in contrast to the psychometric and chronometric curves, the tachometric curve is highly insensitive to the dynamics of the motor planning process. In essence, it reflects how soon (after the cue is revealed) and how strongly the perceptual information modulates motor activity that is already rising. In the context of this urgent decision-making task, both the rising activity—which specifically represents a motor plan—and the threshold are properties intrinsic to the motor circuitry, in agreement with neurophysiological evidence (Costello et al., 2013 see also Hanes and Schall, 1996; Heitz and Schall, 2012). So, variations in threshold could produce a standard trade-off between speed and accuracy in the compelled-saccade task, but this mechanism would leave the tachometric curve intact.

The response threshold is not the only quantity that may be altered to produce a trade-off. In theory, varying the baseline level of activity would be essentially equivalent (see Bogacz et al., 2010). But beyond that, in the context of urgent-decision tasks in which motor planning starts before perceptual analysis, changes in the mean build-up rate of the developing motor activity (Figure 2E) would produce qualitatively similar effects (Figures 2F–H). The intuition is simple: when motor plans rise more quickly, the excursion from baseline to threshold takes less time and there is, consequently, less opportunity for the perceptual information to influence those ongoing motor plans. This case would again correspond to behavioral changes driven exclusively by modulations in the dynamics of the motor circuitry.

Finally, consider another hypothetical scenario in which the only difference between three conditions is in the latency with which the visual stimuli, i.e., the go signal (Figure 2I) and the cue, may start informing the motor plans. This latency could depend on multiple factors, such as contrast or alertness, for instance, but regardless of the cause, everything else being equal, a decrease in visual latency would manifest in a very specific way: it would decrease the mean RTs (Figure 2K), because effectively all afferent delays would be shorter; it would produce a leftward shift of the tachometric curve (Figure 2L), indicating that perception starts guiding performance sooner relative to cue onset; and it would have no effect on the observed percentage of correct responses (Figure 2J), because the motor plans would still have the same amount of time to rise before the arrival of the cue information (stated differently, from the point of view of the motor planning circuit, the time elapsed between the arrival of the go signal and the arrival of the color cue would not change). So, in this case the RT would drop without a trade-off, and the underlying mechanism would be purely sensory/perceptual.

Now, if the mechanisms illustrated in Figure 2 could be combined arbitrarily, it would be possible to produce a range of trade-offs with widely different magnitudes in terms of the ratio of change in percent correct to change in RT. Also keep in mind that, for simplicity, these effects were illustrated based on just three parameters, saccade threshold, mean build-up rate, and visual latency, but various other parameters of the motor and perceptual circuits could serve to modulate performance. So, more generally, different combinations of alterations in the intrinsic dynamics of the motor plans and in the perceptual discrimination process could lead to large or small changes in RT coupled to large or small changes in accuracy.

In conclusion, while overall it may still be true that increases in performance are accompanied by increases in RT, and vice versa, a trade-off may occur for very different reasons, and its magnitude may vary enormously. As will be shown below, this is precisely what appears to be happening under realistic experimental conditions.

## 3. A heuristic modeling framework for describing urgent decisions

This section presents a model that replicates the performance of subjects in the compelled-saccade task and is consistent with the neurophysiology of the underlying neural circuits. Although the model and associated theoretical framework have been described before (for details and parameter values see Salinas et al., 2010; Shankar et al., 2011), they are important for interpreting the experimental data relevant to the speed-accuracy trade-off that are discussed below. We review key findings that establish the model's credibility.

The preparation for action in the context of the compelled-saccade task can be viewed as a competition between two opposing motor plans that develop concurrently, racing to a threshold for triggering a movement to one of the two potential target locations. The direction in which the eyes move is determined by whichever plan reaches the threshold first, but crucially, when perceptual information is available, it modulates the ongoing plans to favor the correct choice. That is the essence of the “accelerated race-to-threshold” model (Salinas et al., 2010; Stanford et al., 2010; Shankar et al., 2011). Such a model is useful because it provides a quantitative, yet intuitive, link between the measured psychophysical behavior and its neural basis.

**Figure 3:**
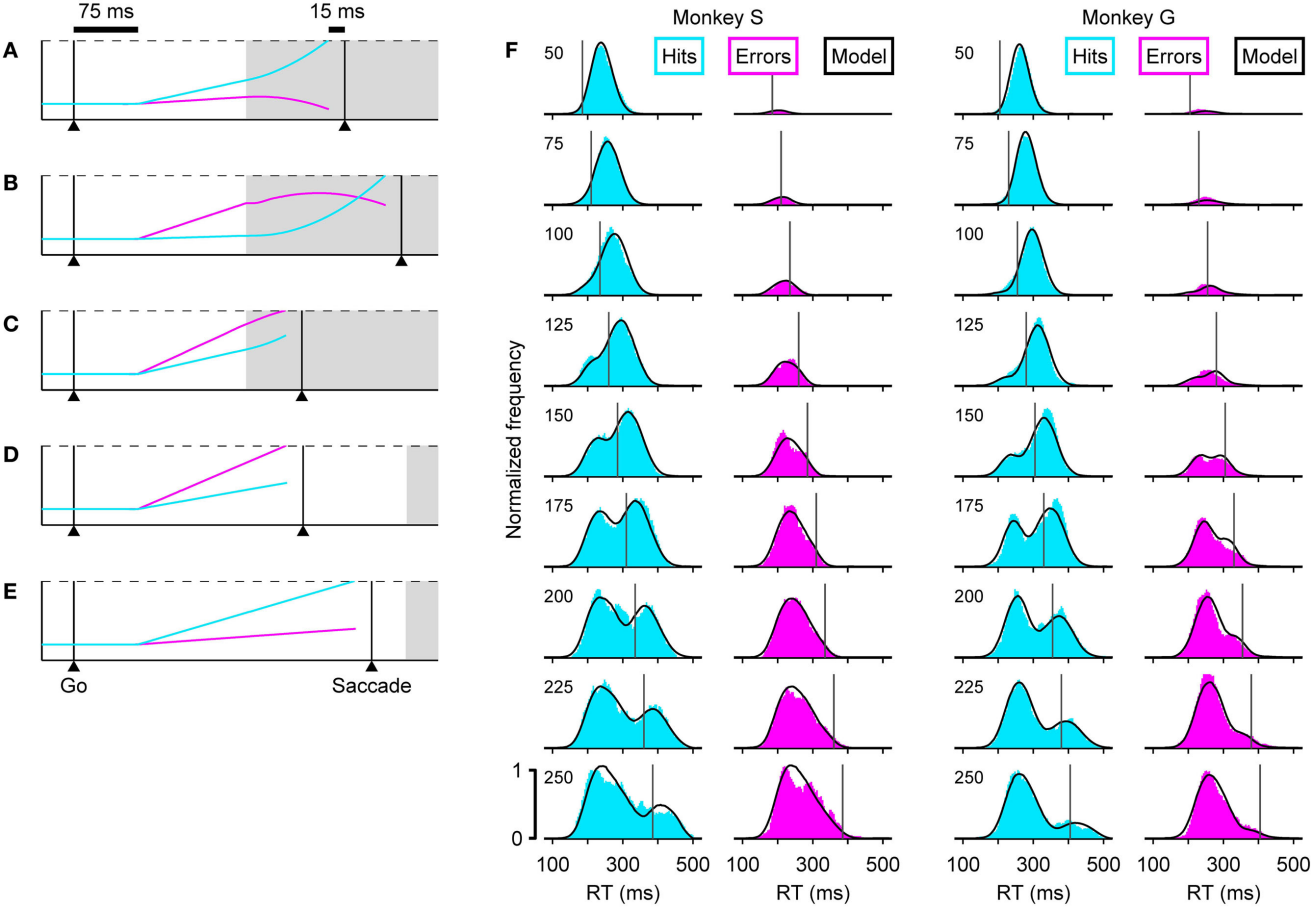
**The accelerated race-to-threshold model closely reproduces psychophysical data in the compelled-saccade task**. **(A–E)** Simulated trials illustrating the five essential types of interaction between competing motor plans. In each panel, two competing variables represent oculomotor activity that triggers an eye movement either to the right (cyan) or to the left (magenta). In these examples the target is on the right, so races in which the cyan trace reaches threshold first correspond to correct choices. The two variables start racing 75 ms (afferent delay) after the go signal and a saccade is triggered 15 ms (efferent delay) after threshold is crossed. Initial build-up rates are drawn randomly in each trial. Gray shades indicate the time during which the cue information is available to modulate the motor activity. During informed choices (**A–C**, gap = 100 ms), the motor plan toward the target accelerates (its build-up rate increases) and the plan toward the distracter decelerates (its build-up rate decreases), whereas during guesses (**D,E**, gap = 250 ms) the build-up rates do not change. **(F)** Reaction time distributions in correct (cyan) and incorrect (magenta) trials at specific gaps (indicated on upper left corners). Results are shown for two monkeys, S (left) and G (right). In each plot, the black curves correspond to model simulations. Vertical lines indicate the center point of the tachometric curve of the corresponding monkey. Results are based on the same experimental and simulated data as in Figure 1.

The dynamics of the model are determined by two key assumptions. (1) That the cue information accelerates the motor plan developing toward the target and decelerates the plan developing toward the distracter. And (2), that in each trial, the competing motor plans begin rising toward threshold shortly after the go signal, with initial build-up rates drawn randomly from a distribution. In this way, the outcome of any given trial depends on when the cue information becomes available relative to how advanced each of the developing oculomotor plans is at that time, and notably, this interaction can take just five distinct forms (Figures 3A–E).

In these examples (Figures 3A–E), a correct choice is produced when the cyan motor plan wins the race. In all trials, the initial build-up rates randomly favor one of the two potential targets, and reflect the subject's initial predisposition. So, when a saccade is triggered before the cue information arrives (Figures 3D,E), the result is an uninformed choice, i.e., a guess. Note that the probability of such a random outcome increases both for longer gap durations and for higher initial build-up rates. In contrast, when the cue information arrives early enough to guide the ongoing motor plans, the result is an informed choice (Figures 3A–C). However, the initial build-up rates still play an important role: when the motor plan that is congruent with the target starts as the leader, it curves upward slightly and triggers a correct saccade with a short RT (Figure 3A), but when this target-related plan lags behind, it starts out slowly and has more ground to cover once the acceleration kicks in, so it takes longer to reach threshold (Figure 3B). On such trials success also requires that the distracter-related plan be decelerated, but if this leading plan is sufficiently advanced, the influence of the cue information may be insufficient to prevent it from reaching threshold and producing an incorrect saccade (Figure 3C).

Thus, the mechanistic signature of the model is this: when the motor activity evoked shortly after the go signal is intense and strongly committed to one of the potential targets, the result is typically an uninformed choice (with a short or negative rPT), whereas when the initial motor activity is more moderate and less biased, the later arriving perceptual information is more likely to resolve the motor conflict in favor of the correct choice (with a long rPT). We think that similar dynamics are, in general, the underlying substrate of rapid perceptual decisions lasting just a fraction of the RT (see Discussion; Cisek and Kalaska, 2010). The accelerated race-to-threshold model, which instantiates these interactions quantitatively, is consistent with both psychophysical and neurophysiological data, as discussed next.

### 3.1. Accounting for the microstructure of behavior

With the correct parameter values, the model can replicate a monkey's psychometric, chronometric and tachometric curves very accurately (Figures 1B–D, compare Data versus Model), but each point in these curves aggregates many trials with motor competitions (races) of different types (Figures 3A–E), so the three curves provide a relatively coarse summary of the subject's behavior. Matching the full RT distributions for correct and error trials at each individual gap (Figure 3F) is a much more stringent benchmark for any model (Salinas et al., 2010), because the shapes of these distributions are directly related to the more limited mixtures of race trajectories that occur at each gap.

For example, the distribution of RTs for correct responses, or hits (Figure 3F, cyan histograms), clearly transitions from unimodal to bimodal. According to the accelerated race-to-threshold model, this is because short gaps contain a large proportion of fast informed decisions (Figure 3A), whereas long gaps contain a mixture of correct guesses (Figure 3E) and slow informed decisions (Figure 3B). Similarly, the distribution of RTs for incorrect responses (Figure 3F, magenta histograms) contains mostly wrong guesses (Figure 3D) and a small proportion of informed choices that were nonetheless incorrect (Figure 3C), which occupy the rightward tail of thehistograms.

These combinations are easy to distinguish by noticing that the rPT that corresponds to the center point of the tachometric curve can be marked as a line in each plot (vertical lines in Figure 3F), and that this line divides each RT distribution into two parts: the trials to the right are all informed choices, whereas the trials to the left are, except for those very near the line, uninformed choices. With this in mind, it becomes immediately obvious that correct trials at short gaps are almost always informed (cyan histograms in Figure 3F, top; RTs are predominantly to the right of the line), whereas correct trials at long gaps are almost always lucky guesses (cyan histograms in Figure 3F, bottom; RTs are predominantly to the left of the line). This also explains why, when looking at the correct responses going from long to short gaps, the peak to the right of the line moves progressively to the left: as the perceptual information arrives earlier and earlier, more and more trials that would have otherwise ended up on the rightward tail of the distribution are accelerated, resulting instead in short RTs. The position of the line itself shifts to the left as the gap decreases because rPT and RT differ precisely by the gap value (Equation 1), but the center point of the tachometric curve remains a fixed number for each monkey—a number that, as mentioned earlier and illustrated in Figure 3, is crucial for assessing the degree to which perceptual information determines the outcome of each trial.

### 3.2. Model parameters and their interpretation

As implemented here, the accelerated race-to-threshold model has 11 parameters that can be adjusted to fit the psychophysical data of individual monkeys, as in Figure 3F (Salinas et al., 2010; Shankar et al., 2011; Costello et al., 2013). Although this number may seem large, the effect of any given parameter is quite limited; each one affects the dynamics of the two competing motor plans in a very specific way and has a well-defined neurophysiological interpretation.

Three parameters describe the distribution from which the initial build-up rates of the two motor plans are drawn in each trial. A description based on three numbers, corresponding to the mean, variance, and correlation of the build-up rates, is quite minimal for a two-dimensional (joint) distribution.

Two parameters, one for the mean and another for the variance, determine the visual latency in each trial. This latency is agnostic about the underlying causes (afferent delay, additional visual processing stages, etc.); it simply describes when the relevant visual information (go and cue) reaches the model circuit. For the results presented here, we assume that the mean latencies of the go signal and the color cue are the same, but this is not necessarily the case in general.

Three parameters describe how perception alters the trajectories of the ongoing motor plans (as in Figures 3A,B); they specify the magnitude of the acceleration and deceleration and how long they last. Using fixed acceleration and deceleration coefficients is the simplest possible way to describe motor plans that are not perfectly straight, i.e., for which the build-up rates are not constant.

One other parameter, the probability of confusion or lapse rate, accounts for incorrect responses that occur at long processing times and cannot be attributed to insufficient cue viewing time. There are many possible reasons for such lapses; here they are simply considered random events.

Finally, two additional parameters are included to replicate a subtle but systematic feature seen in distributions of RTs that are bimodal (as in Figure 3F, for 175–225 ms gap), a dip that is slightly more pronounced than expected. This corresponds closely to a phenomenon known as “saccadic inhibition” that occurs when a distracting stimulus appears while a saccade is already being programmed (Reingold and Stampe, 2002; Buonocore and McIntosh, 2008, 2012; Bompas and Sumner, 2011). The race model accounts for this deviation via a brief interruption in the rise of the motor activity linked to the detection of the cue. The two corresponding parameters determine the onset and offset of the brief pause, and have a relatively minor impact on other aspects of the data.

Thus, the model starts with a simple description of the motor choice process and is augmented with a mechanism whereby perception can guide it. So, is the model activity comparable to saccade-related neural responses evoked during perceptually driven choices?

### 3.3. Linking behavior and neurophysiology

The accelerated race-to-threshold model provides excellent fits to the RT distributions at fixed gaps for all the monkeys we have trained in the compelled-saccade task (Shankar et al., 2011). Although this is certainly reassuring, psychophysical data alone cannot fully constrain or validate such a mechanistic model, even if the fits were perfect; this is true not only for our model (Salinas et al., 2010) but also in general (e.g., Ratcliff and Smith, 2004; Brunton et al., 2013; Miller and Katz, 2013). However, the activity of single neurons recorded in the frontal eye field (**FEF**) of behaving monkeys is consistent with key, non-trivial predictions of the model (Salinas et al., 2010; Stanford et al., 2010; Costello et al., 2013), suggesting that, indeed, its basic layout is correct.

**Figure 4:**
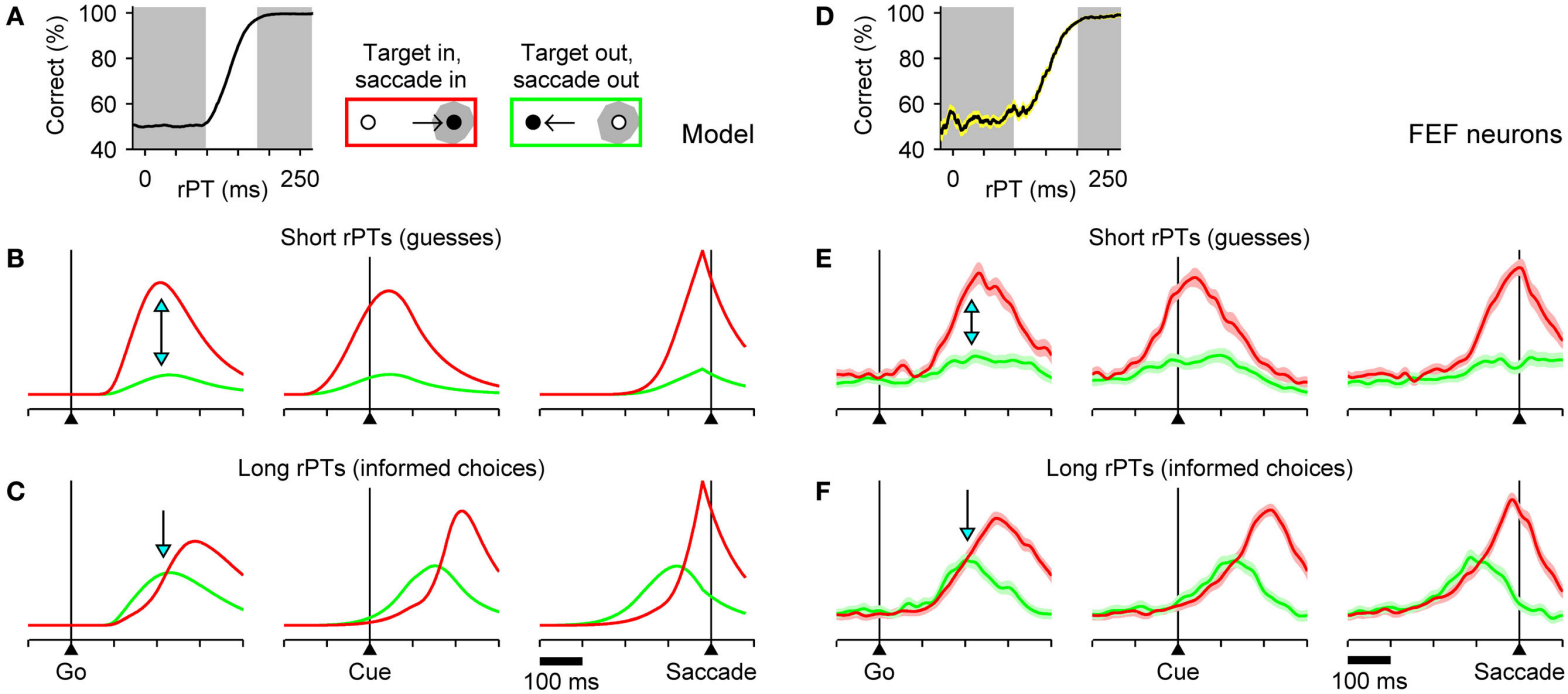
**Comparison between model and FEF neuronal responses**. The accelerated race-to-threshold model was simulated with parameters that fitted the behavioral data of Money S, as in Figure 3, and the simulated activity averaged across trials was compared to that recorded from motor-related neurons in FEF. **(A)** Simulated tachometric curve. Gray shades indicate ranges used to sort the simulated trials into short- (left shade, guesses) and long-rPT (right shade, informed choices) groups. **(B,C)** Average model responses for short- **(B)** and long-rPT **(C)** trials aligned either on the go signal (left column), the cue (middle column), or saccade onset (right column). All data are from correct responses. Separate averages were calculated for choices in the preferred (red traces) and antipreferred (green traces) direction of the model neurons. **(D–F)** As in **A–C**, but for 45 FEF neurons (motor and visuomotor) that differentiated significantly between movements into and away from the movement field before the saccade. Shaded areas indicate ±1 SE across neurons. In all plots, the y axis corresponds to normalized firing rate. Blue arrows mark key differences in evoked activity during guesses versus informed choices. See Costello et al. (2013) for details about the experimental data and modeling methods.

To generate specific predictions directly comparable to neurophysiological data, the model was run with parameter values that fitted the behavioral data of monkey S, and expected neural responses (Figures 4B,C) were computed by averaging separately the simulated motor plans obtained in short- and long-rPT trials. The short- and long-rPT intervals were defined according to the tachometric curve so that they would include chiefly guesses and informed choices, respectively (Figure 4A, shaded areas). In this way we could ask: how should the mean neural responses differ between correct, uninformed guesses and correct, informed discriminations?

The answer to this question comprises essentially two predictions about the relative amounts of activity for saccades in the preferred (red) versus the antipreferred (green) direction of oculomotor neurons. First, during uninformed choices (short rPTs), the motor plan into the movement field should demonstrate a strong advantage shortly after the go signal (Figure 4B; arrows on left column). This preference should be evident before the cue is even presented (Figure 4B; middle column), and corresponds to a heavily biased motor competition that is decided well in advance of saccade onset (Figure 4B; right column). Second, during informed choices (long rPTs), the two motor plans should start building up more slowly and without a strong bias (Figure 4C; arrow on left column). In fact, in this case the expectation is somewhat counterintuitive: during the prolonged period of motor ambivalence, the motor plan in the direction of the target should, on average, lag behind the plan favoring the distracter (red traces below green), but ultimately the conflict must be resolved in favor of the correct choice. The reason for this effect is that, as discussed earlier, correct choices with long rPTs often correspond to trials in which the target-related motor plan is initially weak (Figure 3B), so a similar pattern emerges when averaging over multiple trials (Figure 4C).

The mean evoked responses of FEF neurons (motor and visuomotor) with significant movement-related activity were generally in excellent agreement with the expectations based on the model (Stanford et al., 2010; Costello et al., 2013). In particular, during informed choices, there was, indeed, a prolonged period of motor conflict during which the plan in favor of the distracter showed a slight initial advantage (Figure 4F), whereas no ambiguity was seen during correct guesses (Figure 4E). Observed differences between correct and incorrect responses were also in agreement with the model (Costello et al., 2013). Finally, to compare the model and recorded responses quantitatively, mean traces were calculated and analyzed as continuous functions of rPT via a sliding window, and the ensuing results led to two additional conclusions: (1) that the motor plans favoring the target and the distracter do accelerate and decelerate, respectively, and (2) that the acceleration and deceleration vary as functions of cue viewing time (rPT) as expected given the center point of the tachometric curve (Stanford et al., 2010; Costello et al., 2013).

These results are extremely important because they support the two fundamental elements of the accelerated race-to-threshold model. First, that in the compelled-saccade task, ongoing motor plans are modulated by perceptual information if and when that information becomes available to the motor circuitry, but a motor choice is made either with (informed) or without it (uninformed). And second, that in spite of a profound impact on behavioral performance, the effect of perception on neural activity is rather subtle, particularly for eye movements into the receptive field of the neurons, because acceleration manifests as a slight difference in the curvature of the motor plan as it rises to threshold (Figures 4E,F, right column; compare red traces). Note that it did not have to be this way, as the psychophysical data alone can be replicated very accurately by a model based on completely different assumptions and dynamics (Salinas et al., 2010).

## 4. A trade-off driven by motivational bias

The tachometric curve is highly sensitive to task manipulations (Shankar et al., 2011; Hauser et al., 2013). Thus, many effects—for instance, subtle changes in performance due to perceptual learning (Shankar et al., 2011)—are clearly seen that would normally be impossible to resolve from the raw chronometric and psychometric data. In this section and the next we exploit this to discern, from the results of two experiments, the possible underlying mechanisms whereby speed and accuracy may be traded.

The first experiment consisted of a variant of the compelled-saccade task in which the monkey knew at the beginning of each trial whether a large or a small reward was at stake (all details are described by Shankar et al., 2011). The color of the target conveyed this information, and the association between color and reward amount was kept constant for blocks of 150–250 trials. So, during a block, correct movements to the red target would yield a higher reward than correct movements to the green target, but the high- and low-reward colors were reversed in the next block. Here, because the color of the fixation spot indicates the color of the target, in each trial the subject knows how much reward can be gained, but that is all: given that target color and target location vary randomly and independently across trials, this knowledge provides no objective advantage, although it should affect the subject's motivation to perform the task correctly.

**Figure 5:**
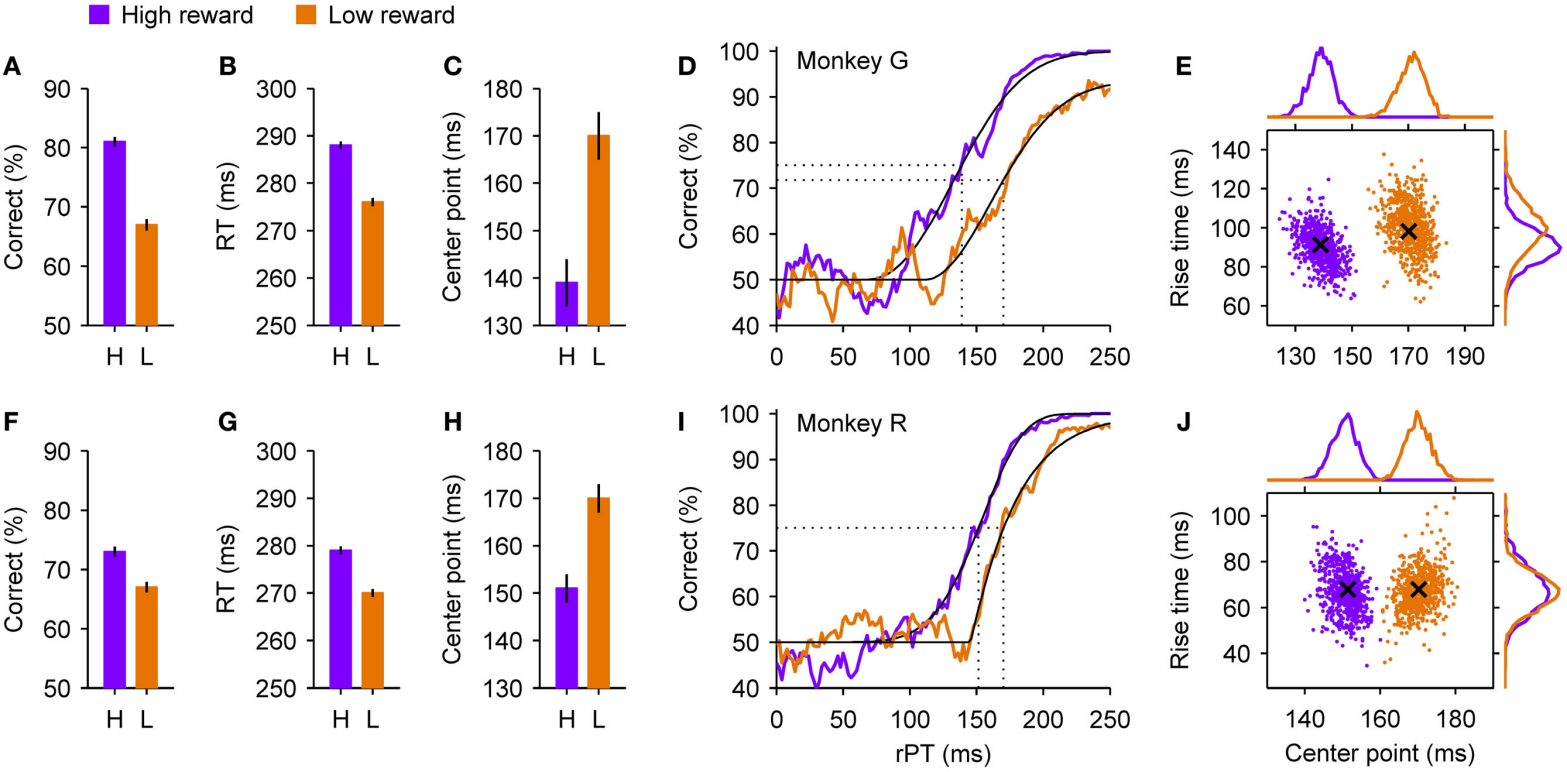
**Psychophysical performance of two monkeys in a motivational bias experiment**. At the beginning of each trial of the compelled-saccade task, the monkey knew whether a correct response would result in a small or a large reward. The shown data were sorted *post hoc* according to the reward that was at stake in each trial, as indicated. **(A–C)** Summary statistics for monkey G. When a high reward (purple) rather than a low reward (orange) was at stake, the overall success rate **(A)** and mean RT **(B)** increased, and the tachometric curve shifted to the left **(C)**, indicating an earlier onset of the perceptual discrimination. Error bars indicate ±1 SE. **(D)** Tachometric curves from monkey G. Fitted Weibull functions (black curves) are shown together with the experimental data (colored traces). A vertical dotted line marks the center point of each curve (indicated in **C**) derived from the fit, i.e., the time point at which the percent correct is halfway between chance and the maximum value. **(E)** Joint distributions of center points and rise times obtained from bootstrapping and re-fitting of monkey G's data, based on 2000 resamplings. The rise time is the time that it would take for the curve to go from 50% to 100% correct if its slope were always equal to the slope at the center point. Crosses mark the values of the original fits shown in **(D)**. Histograms at the top and on the right show the corresponding marginal distributions. **(F–J)** As in **(A–E)** but for monkey R. See Shankar et al. (2011) for details about experimental and statistical methods.

Comparison of responses in the high- and low-reward conditions revealed what appeared to be a classic trade-off between speed and accuracy. When working for a large reward (high incentive), on average the monkeys performed better (Figures 5A,F) and responded more slowly (Figures 5B,G) than when a small reward was at stake (low incentive). Both effects were relatively moderate in absolute terms, but the gain of the trade-off was high: an increase in performance of roughly 10% was accompanied by an increase in RT on the order of 10 ms—a change in RT that is quite small as a fraction of its mean value (~4%). So, based on these data alone, it would seem that the increase in performance incurred a very small cost in RT, and that the system is such that a small flexibility in RT affords a large benefit in success rate. Interpreted in terms of the two motor mechanisms discussed earlier, this would mean that a tiny increase in threshold (Figures 2A–D) or a tiny decrease in the mean build-up rates (Figures 2E–H) would allow the sensory information to have a considerably stronger influence on the outcome of each trial.

However, analysis of the data in terms of processing time paints a much more nuanced picture in which both motor and perceptual mechanisms vary across conditions. In trials in which a high reward was at stake, the tachometric curves of monkeys G and R (Figures 5D,I) shifted to the left by about 30 and 20 ms, respectively, relative to when a low reward was at stake. This suggests that the decision-making process itself starts sooner or advances more rapidly when the incentive to perform accurately is high. By fitting the empirical tachometric curves to continuous functions (Figures 5D,I, thin black lines) and applying resampling techniques to estimate the likely error in these fits (Figures 5E,J), we found that the shifts were very highly significant (Shankar et al., 2011). A leftward shift, however, does not necessarily imply a higher percentage of correct responses, as illustrated earlier (Figure 2J), and would likely be accompanied by *lower* RTs too (Figure 2K), the opposite of the observed effect. So why the discrepancy?

Intuitively, the answer is that at least two mechanisms must be at work across conditions, given that the chronometric and tachometric curves are highly independent. A faster onset of the perceptual process could account for the leftward shift of the tachometric curve, a slow-down in motor activity could account for the increase in performance, and the net effect on RT could be a combination of both.

The accelerated race-to-threshold model confirmed this intuition quantitatively. The model reproduced all the observed effects very accurately, and although this required modifying all of its parameters to various degrees across the two conditions, notably, these parameter differences were qualitatively the same for three monkeys. As discussed earlier, some of the parameters in the model relate fundamentally to perceptual processing and the tachometric curve (e.g., visual latency; magnitude of acceleration/deceleration), whereas others impact the initial motor plans only (e.g., mean and variance of the initial build-up rates). To tease apart their individual contributions to the observed experimental results (Figure 5), we first ran the model that fitted the low-reward condition and then compared the results to those of additional runs in which only selected parameters were modified as required to fit the high-reward condition.

The results were clear: although all parameters changed across conditions and had some impact, the experimental data could be largely explained by the two mechanisms illustrated in Figures 2E–L acting simultaneously. Specifically, according to the model, motor activity developed considerably more slowly during high- than low-reward trials. This slow-down accounted for virtually the full increase in the percentage of correct responses, and in the case of monkey G, if acting alone it would have yielded an increase in mean RT of ~35 ms. This tendency, however, was largely offset by a smaller value of the visual latency parameter that determines when the go signal and the color cue start informing the motor circuit. This change explained most of the shift of the tachometric curve and, by itself, would have produced a drop in mean RT of ~30 ms. So, motor and perceptual mechanisms exerted independent effects on accuracy but opposing effects on speed. As a consequence, the net change in RT produced by the model, with the contributions of all parameters taken into consideration, was relatively small, ~10 ms, the same as found experimentally.

This simple computational dissection indicates (1) that multiple, distinct neural mechanisms are required to simultaneously explain all the experimental findings in the motivation experiment, and (2) that the coincident changes measured in speed (RT) and accuracy (percent correct) do not reflect a single, fundamental trade-off, but rather the combined action of cognitive factors on separate motor and perceptual processes.

Additional experimental observations supported these conclusions. For instance, note that the maximum percent correct reached by the tachometric curves (Figures 5D,I) was not the same in the two conditions. This means that, during trials in which a large reward was on offer, the monkeys rarely made a mistake when provided ample time to discriminate target from distracter, whereas in trials in which the potential reward was small, the monkeys made many more “careless” mistakes, errors that could not be attributed to insufficient viewing time. The frequency with which such errors occur is captured by one model parameter, the lapse rate, and when target and distracter are easily discriminable, as in the experiment, its effect is rather unique—it cannot be reproduced or even approximated by other combinations of parameters—which suggests that it involves yet another mechanism that is distinct from those discussed above.

Therefore, to restate the main conclusion of this experiment, motivation affects choice behavior by simultaneously altering speed and accuracy, and there is good reason to believe that the cognitive signals that mediate these effects are diverse and exert at least partially independent control over motor and perceptual processes (see Discussion). This suggests that, in general, one-parameter descriptions of the speed-accuracy trade-off are likely to be oversimplifications, and should be interpreted with great caution.

## 5. A trade-off driven by spatial bias

Next, we consider a second experiment with asymmetric rewards in the compelled-saccade task. It provides an interesting counterpoint to that in the previous section because it shows that the same motor and perceptual mechanisms may be engaged quite differently across tasks, giving rise to stronger or weaker trade-offs.

In this case, the monkeys received a large reward following correct saccades to one side and a small reward following correct saccades to the other (all details are described by Stanford et al., 2010). As a consequence, they developed a spatial bias, a strong tendency to respond more often to one side than the other. On average, the two animals that participated in this experiment chose the high-reward side about 76% of the time (but this number understates the strength of the preference; see below). The high-reward side, left or right, was kept constant during a block of 150–250 trials and was then switched. As always, target colors and locations were randomly interleaved. The collected data were then sorted according to the subject's choices; that is, trials were partitioned into two groups, those that resulted in movements in the preferred (high-reward) direction, and those that resulted in movements in the non-preferred (low-reward) direction. These two data subsets were then analyzed separately.

**Figure 6:**
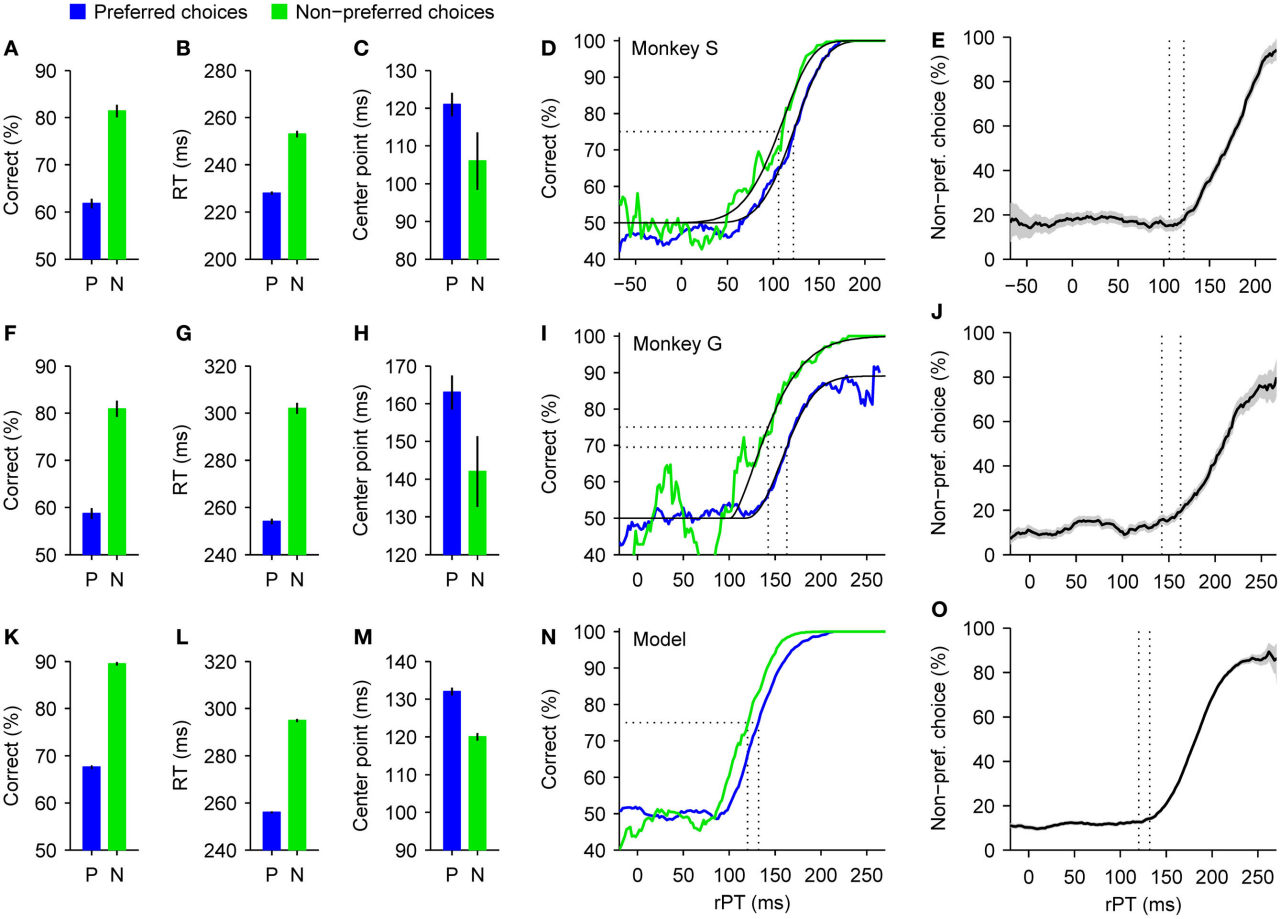
**Psychophysical performance in a spatial bias experiment**. Correct choices to one side yielded a higher reward than correct choices to the other side. Data are shown sorted according to the subject's choices, either to the preferred (high-reward, blue) or the non-preferred (low-reward, green) side, as indicated. **(A–C)** Summary statistics for monkey S. When the preferred rather than the non-preferred side was chosen, the overall success rate **(A)** and mean RT **(B)** decreased substantially, and the tachometric curve shifted slightly to the right **(C)**. Error bars indicate ±1 SE. **(D)** Tachometric curves from monkey S. Fitted Weibull functions (black curves) are shown together with the experimental data (colored traces). Vertical dotted lines mark the center points of the curves (indicated in **C**) derived from the fits. **(E)** Percentage of choices to the non-preferred side as a function of rPT. As in **(D)**, dotted lines mark the center points of the tachometric curves. Gray shades indicate ±1 SE based on binomial statistics. **(F–J)** As in **(A–E)** but for monkey G. **(K–O)** As in **(A–E)** but for simulated responses. Model data were generated with the same parameters as in Figure 4, except that the higher of the two initial build-up rates was assigned to the preferred side in 90% of the trials (instead of the standard 50%). See Stanford et al. (2010) for experimental details.

The behavior of the animals was strikingly different for the two types of choice. Responses in the preferred direction were much more prone to errors than those in the non-preferred direction (Figures 6A,F), and were also initiated much sooner (Figures 6B,G). In other words, the spatial bias induced a trade-off between speed and accuracy across conditions whereby an increase in performance of approximately 20% was accompanied by an increase in RT of 25 or 48 ms, depending on the subject. This behavior can be intuitively understood as follows: the high-reward side is chosen by default, so many choices toward that side are incorrect; in contrast, the low-reward side is chosen only if there is little uncertainty that the target is actually there, but this happens only when the red and green spots are discriminated accurately, i.e., when the rPT (and thus the RT) is long. This can be seen quantitatively by plotting the fraction of choices made to the low-reward side as a function of rPT (Figures 6E,J). The resulting choice curve rises quite sharply, so the monkey's preference is indeed dictated by the amount of cue viewing time. This curve also shows that, in the absence of sensory evidence (rPT ≲100 ms), the monkey's guess is to the high-reward side between 80% and 90% of the time.

In general, the effects on speed and accuracy (Figures 6A,B,F,G) were considerably larger than in the motivational bias experiment (Figures 5A,B,F,G). Interestingly, however, although the main effect on the tachometric curve in this case was again a leftward shift congruent with the condition with higher overall performance (Figures 6C,D,H,I), the magnitude of the shift was smaller than in the motivational bias experiment (Figures 5C,D,H,I). This suggests that the perceptual process itself was affected less by the spatial bias than by the motivational bias, and therefore, that the observed trade-off in the former case may be accounted for almost entirely by an internal adjustment in motor planning alone. Indeed, that is precisely what a more thorough analysis of the data showed.

Again we used the accelerated race-to-threshold model to estimate the contributions of different mechanisms to the biases found empirically. However, instead of discussing the full model fits to the psychophysical data, which involve numerous parameter differences across conditions, in this case we discuss a much simpler manipulation that illustrates the main result more plainly. It goes as follows. First we simulated *N* trials of the model with a fixed set of parameters. This set was exactly the same one used earlier to reproduce the behavior of monkey S (Figure 3F); everything was balanced, unbiased. Then we divided the simulated trials into two groups with approximately *N*/2 trials each: one group included all the trials in which the motor plan to the right had led initially, before the cue information was presented, and the other group included all other trials, in which the plan to the left had drawn a higher initial build-up rate. For this, trial outcome was irrelevant; only the initial build-up rates were considered. Next, we designated the right side as the preferred, highly-rewarded side, and threw away 89% of the trials in the second group, in which the non-preferred (left) plan had led initially. Finally, we merged the remaining simulated trials back into a single data set, erased the information about which plan led initially, and analyzed them exactly as if they had been collected in the experiment. With this method, we produced a biased data set without changing the influence of the perceptual information or the dynamics of the motor plans at all; all we did was create a hypothetical subject, just like monkey S, that made 90% of its initial guesses toward a preferred side (combining *N*/2 preferred guesses with 0.11 × *N*/2 non-preferred guesses makes the former 90% of the total).

When the synthetic trials thus generated were sorted according to choice, as was done with the monkey data, the results qualitatively mimicked all the effects found experimentally: choices in the preferred direction were less accurate (Figure 6K) and faster (Figure 6L), the probability of making a non-preferred choice varied sharply as a function of rPT (Figure 6O), and the tachometric curves derived from preferred and non-preferred choices were slightly shifted relative to each other (Figures 6M,N). The underlying reason why such large differences emerged is that, by selecting trials based on the direction of the leading motor plan, the proportions of the five basic types of motor competition (Figures 3A–E) became drastically different for the two possible choices. Such proportions alone have an enormous impact on the average RT and success rate, even when the dynamics remain identical within each type of race. So, all the relevant differences between preferred and non-preferred choices—and in particular the bulk of the speed-accuracy trade-off—can be explained by a simple asymmetry in the way the motor plans are initially deployed.

This is not to say that other properties of the motor plans or of the perceptual process that informs them remained perfectly intact. In fact, there are hints that they did not. One is that the maximum percent correct was significantly different for the two tachometric curves of monkey G (Figure 6I), and another is that the shifts seen in the real data were larger than that in the simulation (Figures 6D,I,N). Additional adjustments to the parameters of the model would be required to account for these effects. However, these discrepancies are relatively small and do not affect the main conclusion, which is that in the spatial bias experiment the trade-off is larger than in the motivational bias experiment and depends predominantly on the way the motor plans for the two choices are deployed at the beginning of each trial.

Perhaps somewhat counterintuitively, these results also imply that average RTs may decrease in one condition relative to another without any explicit slow-down of the motor circuitry. If this circuitry naturally produces a wide distribution of RTs, then the apparent difference in response speed may result simply because one condition samples more fast and fewer slow trials than the other. In this sense, a change in response speed may not necessarily reflect a change in dynamics.

Taken together, the results reviewed in this and the previous section indicate that the individual contributions of motor and perceptual mechanisms to a given, experimentally observed trade-off may vary widely depending on the particular circumstances of an experiment.

## 6. Broader implications

Here we have reviewed behavioral, neurophysiological and modeling results in an urgent decision-making task in which independent, quantitative measures of motor and perceptual capacity (chronometric and tachometric curves) can be obtained. Based on this unique dissociation, we investigated how motor and perceptual mechanisms interact to determine a subject's response speed (RT) and accuracy (percentage of correct choices). In other words, we were able to decouple these quantities and investigate the potential sources of their trade-off.

Based on a combination of behavioral and neurophysiological constraints, the accelerated race-to-threshold model provides a parsimonious description of how perceptual information may resolve an ongoing motor selection process during relatively rapid choices. This heuristic model is key because it lets us evaluate the functional roles that meaningful neural elements or features play in the choice process. It shows, for instance, that the build-up rates with which competing motor plans are deployed initially, before perceptual information arrives, are absolutely critical in determining the fate of any given task trial (Figure 4, see arrows Salinas et al., 2010; Shankar et al., 2011). Likewise, the tachometric curve demonstrates that the response latencies—neuronal, not behavioral—to the go signal and the cue are much more flexible than one might have expected (Figures 5D,I), and the model serves to evaluate quantitatively the consequences of this (Figures 2I–L; see also Salinas and Stanford, 2013). Of course, other neural parameters may be important too; the point is simply that many specific properties of perceptual and motor-planning circuits may be quantitatively related to simultaneous changes in speed and accuracy.

When seen under the light of this framework, the experimental results obtained in the two biased versions of the compelled-saccade task lead to three conclusions: (1) that both motor and perceptual mechanisms may contribute to an observed trade-off, (2) that each of these mechanisms may weigh in more or less heavily, depending on the particulars of the task, and (3) that, as a consequence, small or large trade-offs may result from various combinations of motor and perceptual contributions.

This would also explain why, under certain circumstances, it is possible to observe a decrease in RT and/or an increase in accuracy with no apparent trade-off (Bendiksby and Platt, 2006; Takikawa et al., 2002). Other studies are also consistent with an intricate, fluid link between perception and action (Battaglia and Schrater, 2007; Cardoso-Leite and Gorea, 2010; Simoncini et al., 2012; see below).

### 6.1. Life without the tachometric curve

It is interesting to ponder how the two bias experiments would be interpreted without the tachometric curve. In the case of the motivational bias, the trade-off would seem small (Figures 5A,B,F,G), and there would be no reason to think that the perceptual evaluation itself would or should change from one condition to another. The results could be explained as a small increase in a response criterion leading to slightly better performance and slighlty higher RTs. Instead, the tachometric curve reveals significant changes in perceptual performance (Figures 5D,I), and it is only because of the model that those changes can be reconciled with the relatively small observed trade-off, and that a rather substantial adjustment in motor planning can be inferred.

In the spatial bias experiment the speed-accuracy trade-off is large and evident (Figures 6A,B,F,G), but without the tachometric curve it again would be virtually impossible to ascertain whether or not changes in perception are involved—such changes are there (Figures 6D,I), but are noticeably smaller and less important in proportion to the magnitude of the trade-off in this case. Furthermore, the choice curve (Figures 6E,J) and the model (Figures 6K–O) provide a clear and parsimonious account of the results: the subjects' strategy is to almost always make an initial guess toward the preferred side, and override that initial plan only when the perceptual evidence against it arrives early enough and is strong enough. Without this insight, which depends critically on the distinction between RT and rPT, it would be very difficult to understand why, at a given gap, the subjects choose the low-reward side on some trials but not on others.

Interestingly, if the goal of the internal circuitry is to implement said strategy, then the observed trade-off may be a plain byproduct of the implementation, because the results can be largely accounted for simply by appropriately redistributing simulated trials across conditions, without altering any parameters or interactions in the model. In other words, the internal circuitry may not be directly attempting to find an optimal compromise in the exchange of RT for percent correct; rather, the observed exchange may be an inevitable consequence of a different trade-off, that between the possibility of a large reward versus the certainty of a small one.

### 6.2. Ubiquity of fast decisions

A few other tasks used in past studies compel participants to make a response before the correct answer is fully specified (Schouten and Bekker, 1967; Becker and Jürgens, 1979; Ghez et al., 1989; Hening et al., 1998; Chapman et al., 2010; Wood et al., 2011). In particular, the countermanding or stop-signal task is very similar to our compelled-saccade task, except for two main differences: it is a go/no-go task, and the relevant sensory evaluation is a detection rather than a discrimination—but notably, a tachometric curve can be constructed in this case too (Salinas and Stanford, 2013). Numerous experimental manipulations of the countermanding task have led to simultaneous changes in RT and percent correct (Cabel et al., 2000; Cavina-Pratesi et al., 2001; Ramautar et al., 2004; Emeric et al., 2007; Stevenson et al., 2009; Leotti and Wager, 2010), and modeling work indicates that, in different experiments, the observed trade-off may result either from adjustments in motor planning alone, in the perceptual detection process alone, or in both (Salinas and Stanford, 2013). The parallels with the experiments reviewed here are striking. For instance, variations in response latency associated with the detection of the saccadic target and the stop signal seem to be major determinants of perceptual performance. Overall, the spectrum of potential speed-accuracy trade-offs in the countermanding task is just as wide as illustrated here, if not wider, in terms of their magnitude and variety of underlying neural mechanisms (Salinas and Stanford, 2013).

These results notwithstanding, how general are the conclusions presented here? Perhaps compelled-response tasks put subjects in an unnatural setting in which the mechanisms that control speed and accuracy are engaged in rather anomalous ways. To the contrary, we think that compelled tasks are good models for many real-life situations in which choices are made quickly (see Uchida et al., 2006).

For instance, eye movements (2–3/s) show similar distributions of fixation times and intersaccadic intervals under a wide variety of viewing conditions (Berg et al., 2009; Castelhano et al., 2009), which suggests that they are normally programmed continuously, without waiting for particular perceptual events to happen (McPeek et al., 2000; Hafed and Ignashchenkova, 2013). Furthermore, the ability to quickly modify ongoing motor plans is essential in situations that demand extreme performance, such as high-speed chases (Ghose et al., 2006, 2009). Competitive sports provide many familiar examples too. To return a tennis serve, hit a curveball, or stop a penalty, movements must be prepared early and the corresponding motor plans must take into account relevant perceptual information as soon as it becomes available (Abernethy, 1990; Land and McLeod, 2000; Yarrow et al., 2009). Interestingly, athletic skill may be thought of as an unusually weak speed-accuracy trade-off, in that a professional squash player can strike the ball both faster and more accurately than a beginner, and there is evidence that when the skill level achieved is exceptional, it is so in both perceptual and motor domains (Yarrow et al., 2009).

In this respect, note that the “urgency” of the compelled-saccade task refers to the perceptual analysis process rather than to motor execution. The saccadic RTs obtained in the task (Figure 1C) are well within the normal range for choice behaviors (e.g., DiCarlo and Maunsell, 2005; Berg et al., 2009); it is the color discrimination that is time-limited. For a participant, the decision is urgent in the same way as for a batter trying to hit a baseball: there is ample time to perform a required movement (a saccade or a swing), but very little time to make the relevant judgment (red/green or curveball/fastball) *and* inform the ongoing motor plan so that the movement is correct. In contrast, by specifically requiring that subjects remain still while the critical sensory information is displayed, the majority of laboratory tasks used to study perceptual decision making abolish this temporal conflict, both in fixed-duration and RT paradigms. This, however, makes it extremely difficult to determine when the perceptual discrimination finishes and when the motor plans start (e.g., Kiani et al., 2008; Port and Wurtz, 2009; Zariwala et al., 2013)—and thus to attribute a given change in mean RT to either of these events.

### 6.3. Urgent versus non-urgent decisions

The distinction between urgent and non-urgent tasks parallels a broad conceptual division in the ways in which sensory, cognitive and motor circuits may interact to carry out goal-directed actions or choices. In one scenario, they operate in a strictly serial fashion whereby perceptual analysis needs to reach a conclusion first, before the motor selection process can begin. In the alternative scenario, the simultaneous activation of multiple uninformed motor plans marks the start of the choice process, and the competition is subsequently guided by perceptual information on the fly, if and whenever it becomes available. Each of these possibilities is likely to apply under certain circumstances. Cisek and Kalaska (2010), Cisek (2012) and Padoa-Schioppa (2011) discuss this issue at length. Here, we make two observations about this distinction in regard to our results.

First, we note that the former, serial account is incompatible with the compelled-saccade task (Salinas et al., 2010), but beyond that, one could argue that, for time scales below roughly 1000 ms, the idea of sequentially ordered perceptual and motor stages is inconsistent even with results from ostensibly serial decision tasks. This can be appreciated in two limit cases in which the trade-off between speed and accuracy essentially disappears. At one extreme, performance in many tasks does not benefit from prolonged deliberation times beyond 250–300 ms (Uchida et al., 2006), so that the optimal behavior is to respond rapidly (within <300 ms) regardless of difficulty. This is precisely what Mainen and colleagues found in an odor categorization task in rats (Zariwala et al., 2013). At the other extreme, note that the rise in choice-related firing activity is often interpreted as a pure accumulation of sensory evidence (Gold and Shadlen, 2001), but the notion that sensory evidence must achieve a critical threshold before the effector system is engaged is difficult to reconcile with choices made on the basis of little or no sensory evidence. Consider, for instance, the zero-coherence trials in the random-dot motion discrimination task (Shadlen and Newsome, 2001); what drives choice commitment when the sensory input to be integrated consists exclusively of noise? A choice under such condition is typically framed and modeled as the result of a lower threshold or collapsed decision bound wherein the evidence criterion is relaxed so that less (or no) sensory evidence is required to engage the motor circuitry (Ditterich, 2006; Beck et al., 2008; Hanks et al., 2011). But this is essentially a matter of interpretation: a collapsing decision bound is functionally equivalent to an increasing motor plan or urgency signal (Cisek et al., 2009; Thura et al., 2012). So, viewed from a different perspective, the “perceptual threshold” can be interpreted as the point in time at which a commitment to a motor choice curtails the evidence accumulation phase that had been informing the emerging motor plan to that point. Importantly, current neurophysiological evidence (Hanes and Schall, 1996; Heitz and Schall, 2012; see also Hayden et al., 2011) indicates that there is indeed such thing as threshold crossing, at least for saccadic choices, but it is a decidedly motor event. Furthermore, as the choice-related activity rises, its level relative to threshold is directly related to the degree of motor commitment (Gold and Shadlen, 2000).

Second, several studies within the latter camp, which considers the scheduling of motor actions to be independent of perceptual events, resonate particularly well with our approach. In particular, Goodale and colleagues studied the hand trajectories that result when humans perform a compelled-reaching task (Chapman et al., 2010; Wood et al., 2011). Participants were obliged to begin execution of a pointing movement toward one of various stimuli, but information identifying the true target was released only after the onset of the reach. The characteristic spatial patterns that resulted indicated that, initially, multiple reaching plans toward various potential targets develop in parallel, with the initial movement direction reflecting an underlying vector-averaging operation; the final movement direction is disambiguated later, when the true target is revealed. Interestingly, they also found that stimuli of greater salience (through greater contrast or pixel density) confers greater initial weight to their corresponding motor plans, even when such saliency is unlikely to signal the true target location (Wood et al., 2011; see also Schütz et al., 2012). Notably, this pop-out effect went away when participants were allowed to briefly view the stimulus cue before initiating the reach. This means that motor plans associated with salient stimuli are activated more strongly, but unless the observer has reason to believe that a stimulus is important beyond its perceptual salience, this increased weight dwindles rapidly. So, perceptual information continuously modulates ongoing motor plans, likely via multiple pathways (e.g., bottom-up versus top-down).

In agreement with the aforementioned findings in FEF (Stanford et al., 2010; Costello et al., 2013), this conclusion is highly consistent with analyses of single-neuron activity recorded in the parietal reach region of monkeys, which show (1) that competing motor plans are initially activated when multiple reach targets are presented and a choice needs to be made (Scherberger and Andersen, 2007), and (2) that the motor conflict is resolved either spontaneously or once the relevant cue information is provided (Klaes et al., 2011). Similar ideas have also been advocated by Cisek and colleagues based on recordings from premotor areas (Cisek and Kalaska, 2005; Pastor-Bernier and Cisek, 2011), giving rise to a powerful modeling framework, the “urgency-gating model” (Cisek, 2006; Cisek et al., 2009; Thura et al., 2012), that is similar in spirit to our accelerated race-to-threshold model (see Costello et al., 2013).

These findings demonstrate that, during rapid choices, perceptual and motor planning processes overlap extensively in time and are likely to contribute jointly to RT and accuracy under many circumstances. Their interaction is evident even in the absence of motor competition, when the upcoming movement is certain (Buonocore and McIntosh, 2008, 2012; Welchman et al., 2010; Bompas and Sumner, 2011). As a consequence, pinpointing the mechanisms that give rise to an observed trade-off is likely to be exceedingly difficult in general—unless additional experimental constraints independent of RT and percent correct are available.

### 6.4. Back to the future: a historical note

The existence of a speed-accuracy trade-off has been acknowledged for many years (Woodworth, 1899; Hick, 1952), and it once was considered to have “great potential to advance all areas of cognitive psychology” (Wickelgren, 1977).

In 1977, Wickelgren passionately argued that generating speed-accuracy functions—the curves obtained by plotting the percentage of correct responses versus RT—would be vastly superior to simply evaluating RT and performance in single, independent experiments. He reasoned that a prototypical speed-accuracy curve would have three essential components: (1) an initial delay period during which performance would be at chance, (2) a ceiling value reached at long RTs beyond which performance could not increase further, and (3) a steep rise in performance around a short window of RTs. All three features would be informative and potentially interpretable in terms of separate cognitive mechanisms. Wickelgren (1977) further distinguished two ways to create such a curve, both potentially useful. One version used the “macro-trade-off,” which is what commonly results when experimental manipulations are introduced (i.e., via deadlines, asymmetric payoffs, instructions emphasizing speed or accuracy, etc.); the other version used the “micro-trade-off,” which is seen by the *post hoc* partitioning of RTs from a single experiment into small bands for analysis. Building on the work of Pachella (1974), Wickelgren suggested that internal variations in response criteria due to arousal, attention, and other covert factors creates variability within the RT distribution that macro-plots might not account for, but that would manifest in the micro-curves.

**Figure 7:**
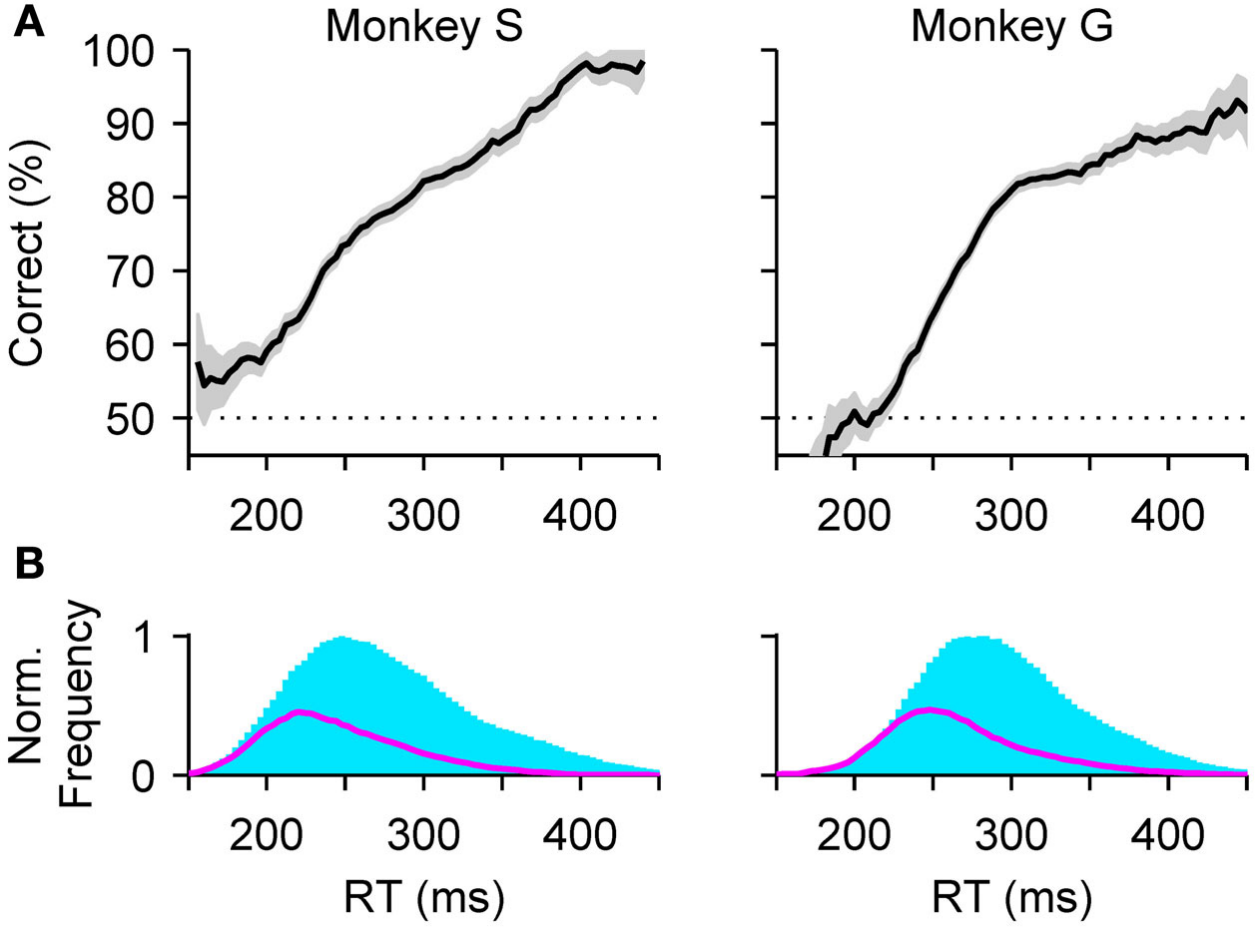
**Speed-accuracy curves in the compelled-saccade task**. **(A)** Percentage of correct responses as a function of RT for monkeys S (left) and G (right), computed from the same experimental data shown in Figure 1. Trials were sorted according to RT, regardless of gap, using bins with a 40 ms width sliding in steps of 2 ms. Gray shades indicate ±1 SE based on binomial statistics. **(B)** RT distributions for correct (blue) and incorrect (magenta) choices for each monkey, based on the same bins used in **(A)**.

These ideas faded somewhat (but see, e.g., Giordano et al., 2009), most likely, we suspect, because the shapes of the speed-accuracy curves obtained experimentally were not stereotypical, as was hoped initially, nor consistent across experiments. For example, when the curves are generated from the data in the standard compelled-saccade task (Figure 7A), the resulting shapes are essentially meaningless. The framework presented here makes it easy, in retrospect, to see the reason for such failure: RT is not the same thing as processing time, and it is the relationship between performance and *processing time* that is stereotypical. That relationship—which is none other than the tachometric curve—describes precisely how much accuracy is gained for a given amount of time. It does this within a given experiment, as the micro-curve was supposed to do, and also decouples any true changes in perception from purely motor variations in RT, as may occur during a macro-trade-off. For the speed-accuracy curve to work as envisioned, the RT would need to correlate very tightly with rPT, but in general it does not, because it additionally depends on many cognitive processes such as attention, memory, or motor planning, that contribute to its variance (Figure 7B).

Wickelgren (1977) recognized the enormous utility of a curve that would accurately reveal the dependence of performance on time. It could serve as a powerful tool for studying the dynamics of information processing across subjects, modalities, and task conditions, and by extension, for studying the neural mechanisms underlying fundamental cognitive functions. We submit that it is the tachometric curve, not the speed-accuracy curve, that fulfills this promise.

### Conflict of interest statement

The authors declare that the research was conducted in the absence of any commercial or financial relationships that could be construed as a potential conflict of interest.
